# What Is the Correlation between Clinical and Radiographic Findings in Patients with Advanced Osteoarthritis of the Knee?

**DOI:** 10.3390/jcm12165420

**Published:** 2023-08-21

**Authors:** Moritz M. Innmann, Andre Lunz, Larissa Fröhlich, Thomas Bruckner, Christian Merle, Tobias Reiner, Marcus Schiltenwolf

**Affiliations:** 1 Department of Orthopaedics, Heidelberg University Hospital, Schlierbacher Landstrasse 200a, 69118 Heidelberg, Germany; 2 Institute of Medical Biometry and Informatics, University of Heidelberg, Im Neuenheimer Feld 305, 69120 Heidelberg, Germany; 3 Diakonie Klinikum Stuttgart, Rosenbergstraße 38, 70176 Stuttgart, Germany

**Keywords:** osteoarthritis, knee arthroplasty, range of motion, pain, PROM, Oxford knee score

## Abstract

Knee range of motion and patient-reported outcome measures (PROMs) are often used as screening tools to assess the severity of knee osteoarthritis and guide the decision to refer patients to an arthroplasty clinic. However, there is little understanding regarding the correlation between these factors. Thus, the purpose of this study was to determine the correlation between patient-reported clinical function measured with the Oxford Knee Score (OKS), pain assessed using the visual analog scale (VAS), knee range of motion (ROM), and characteristic radiographic features in patients with advanced osteoarthritis of the knee. A prospective analysis of a consecutive series of 138 patients with advanced unilateral osteoarthritis (OA) of the knee was performed. The severity of radiographic OA was classified according to the most commonly used Kellgren and Lawrence classification (K&L). Spearman’s rank correlation analysis and multiple linear regression analysis were performed. The OKS was used as a dependent variable and was adjusted for pain, ROM, and nine standardized radiographic parameters on multiple views of the tibiofemoral and patellofemoral joint. OKS and pain correlated weakly with the K&L grade (r = −0.289; *p* = 0.001; r = 0.258; *p* = 0.002). K&L grade and the degree of patellofemoral joint space narrowing were identified as independent factors being associated with a poorer OKS (coefficient −4.528, *p* = 0.021; coefficient −2.211, *p* = 0.038). Slightly worse results were identified for OKS and pain in patients with K&L grade 4 osteoarthritis compared to patients with K&L grade 3 osteoarthritis (∆OKS 5.5 points, *p* < 0.001; ∆VAS 1.7 points, *p* = 0.003). There was no significant difference for passive range of motion between patients with K&L grade 3 or 4. When counseling patients with advanced knee osteoarthritis who may be eligible for knee arthroplasty, it is essential to give primary consideration to pain levels and self-reported limitations experienced during daily activities. Relying solely on knee ROM and PROMs is not an effective screening method for guiding the decision to refer patients to an arthroplasty clinic.

## 1. Introduction

The number of patients with advanced osteoarthritis (OA) of the knee treated with knee arthroplasty has been constantly increasing in recent years [1,2]. Although methods and numbers of projection vary considerably, estimates for the upcoming years predict a further increase in the demand for primary total knee replacement (ranging from 117% to 673% by 2030) [3,4,5]. Radiologic imaging, clinical examination with a focus on the leg axis, deformity, and range of motion (ROM) of the knee, along with patient-reported outcome measurements (PROMs), are utilized as standard tools for evaluating the overall severity of OA and its impact on daily activities and quality of life. The attending physician provides counseling to patients with OA regarding subsequent treatment options based on the findings described above. However, in many legal cases involving OA in Germany, the severity of OA and impairment (and potential compensation) are classified based on the radiographic degree of OA in the affected joint, rather than the clinical function. This means that a higher degree of radiographic osteoarthritis is assumed to result in worse clinical function for the patient, even though the clinical examination might yield different results. For many joints, a higher degree of radiographic OA is highly correlated with worse clinical function of the joint. However, it is not well understood to what extent this correlation also applies to knee OA. Furthermore, patients are often evaluated for eligibility for total knee arthroplasty based on PROMs before being referred to an arthroplasty clinic. This approach may result in an under- or over-identification of patients who potentially require knee arthroplasty.

Before conducting this study, we performed a comprehensive literature review on the current topic, yielding the following information: A study by Rupprecht et al. detected a significant correlation between the functional score of the International Knee Society (IKS) and the results of the radiographic examination, but not for Western Ontario and McMaster Universities Osteoarthritis Index Score (WOMAC) and pain [6,7]. Özden et al. found that K&L grade showed a strong correlation with VAS pain and patients’ activity [8]. Another study showed significantly worse scores for the Hospital for Special Surgery (HSS) Knee Score according to the Ahlbäck classification of knee osteoarthritis [9]. Duncan et al. reported on an association between severity of pain, stiffness, and physical function and the presence of radiographic signs of OA in a population-based study of 819 adults with knee pain [10]. In another study, Dowsey et al. identified only an association between IKS and the presence of osteophytes of the lateral compartment of the knee [11]. In contrast to the aforementioned studies, Son et al., in a sub-analysis of two patient registries, found that 6% to 31% of patients with K&L grade 4 OA had no pain [12]. Similarly, Steenkamp et al. found no correlation between WOMAC pain and functional scores, patient factors, and radiological severity of OA of the knee in a prospective study cohort on 52 patients [13]. Johnson et al. demonstrated that alterations in WOMAC scores among patients with knee OA were not correlated with changes in PROMs, disease activity, radiographic progression, or subjectively perceived clinical outcomes [14].

In addition to the variable and contradictory findings of the heterogeneously designed studies cited above, it is worth noting that only the last mentioned study included adequate statistical power to identify a clinically significant correlation between groups in terms of the minimal clinically important differences (MCIDs) of the PROMs [14].

In summary, our recent and comprehensive literature review has revealed that only a limited number of well-designed studies have been published and that these studies have provided inconclusive results regarding the associations between PROMs and clinical and radiographic findings in patients with advanced OA of the knee. Therefore, the current exploratory prospective study was conducted to examine the correlation between the Oxford Knee Score and clinical as well as radiological findings in patients suffering from advanced osteoarthritis of the knee. The objective of this study was to investigate the following inquiries: (1) Is there a correlation between the Oxford Knee Score (OKS), pain measured on the visual analog scale (VAS), knee range of motion (ROM), and characteristic radiographic features in patients with advanced knee OA? (2) Can a correlation be established between the OKS and clinical parameters, as well as characteristic radiographic features of knee OA, when considering them as independent variables using multivariate linear regression analysis?

## 2. Materials and Methods

### 2.1. Study Population

**Figure 1:**
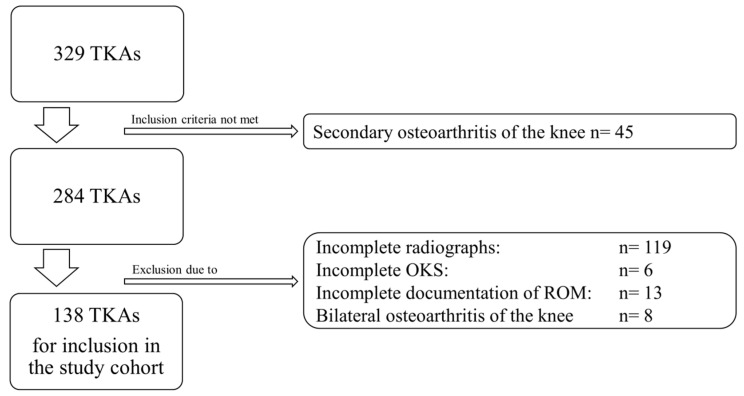
CONSORT flow diagram for identification of the study cohort.

The study cohort consists of a consecutive series of 138 patients (138 knees) with a mean age of 65 years (range, 39–85 years). All patients who presented with knee pain and advanced unilateral primary osteoarthritis of the knee (K&L grade 3 or 4) in the outpatient clinic of our institution in 2019 were included in the study. All patients included in the study had radiographic evidence of knee osteoarthritis (OA), ensuring the exclusion of patients with isolated patellofemoral pain syndrome, which, by definition, lacks structural alterations within the knee. As the study aimed to investigate a potential correlation between the Oxford Knee Score, pain, range of motion, and radiographic features in patients with advanced primary knee OA, we had to exclude patients with incomplete data sets (radiographs, OKS, or ROM). Furthermore, we aimed to investigate the study question in patients with degenerative primary knee OA and no other reasons for OA (e.g., posttraumatic OA) in order to maintain a homogenous study cohort. Patients with bilateral advanced osteoarthritis of the knee were also excluded because knee OA of the other side affects the OKS and might bias the results. Thus, the exclusion criteria were as follows: secondary osteoarthritis (*n* = 45), missing or incomplete radiographs (*n* = 119), incomplete documentation of ROM (*n* = 13), incomplete PROMs (*n* = 6), or bilateral advanced osteoarthritis of the knee (*n* = 8) (Figure 1). The study was approved by the institutional ethics board on 4 January 2016 (Nr. S-620/2015) and was conducted in accordance with the Helsinki Declaration of 1975, as revised in 2013. All data were routinely collected upon the admission of each patient and recorded in our institutional database.

### 2.2. Patient Demographics and PROMs

Patient demographics are summarized in Table 1. The data sets were exported from the institutional database after pseudonymization into Microsoft Excel (Microsoft Excel 2019 for Windows, Microsoft Corporation, Santa Rosa, CA, USA) for further data evaluation. Patient data comprised of age, gender, body mass index (BMI), American Society of Anesthesiologists (ASA) status, diagnosis, knee pain, contralateral knee pain, Oxford Knee Score (OKS), and passive ROM of the affected knee. The range of motion (ROM) of the affected knee was assessed during a physical examination conducted by an orthopedic surgeon at the outpatient clinic. The evaluation involved utilizing a goniometer with 1-degree increments to measure the precise angles. Pain of the affected and contralateral knee was graded by the patients on a visual analogue scale (VAS) that ranged from 0 to 10 (0 = no pain, 10 = maximal pain). The OKS is a standardized PROM that has been validated for use in patients with OA of the knee. It is a 12-question assessment that yields a score ranging from 0 (indicating the lowest performance) to 48 (indicating the highest performance). The score reflects the patient’s perspective of the function of the knee during their activities of daily living before or after knee replacement. It was initially developed to assess outcomes after knee arthroplasty and was designed to be completed by the patient in order to minimize potential bias unwittingly introduced by medical staff when assessing the results themselves [15]. The OKS has several advantages when compared with other PROMs. It is a concise questionnaire consisting of only 12 questions, making it relatively quick and easy to administer. This is particularly beneficial in clinical settings where time constraints are an important factor. Other widely used PROMs like the WOMAC (Western Ontario and McMaster Universities Arthritis Index) or KOOS (Knee injury and Osteoarthritis Outcome Score) contain more items (24 and 23, respectively) and are therefore more time-consuming to complete. Furthermore, the OKS is assessed from the patient’s perspective and it can be assessed remotely by post, over the phone, or online. Therefore, it has been described that the score might be used as a screening tool for knee OA patients in order to assess an individual patient’s suitability for knee replacement surgery [16].

### 2.3. Radiographic Parameters and Analysis

Various standardized digital radiographs were taken for all patients and stored in our institutional Picture Archive and Communications System (PACS, Centricity, GE Healthcare, in Chalfont St Giles, UK). These radiographs include the full weight-bearing anteroposterior (a.p.) and lateral views of the knee, as well as the full weight-bearing long leg a.p. view and a skyline view of the patellofemoral joint. Radiographic analysis was performed in a prospective manner for every patient by two independent reviewers blinded to each other (MMI, LF) and blinded to other radiograph images of the included patients to prevent the risk of potential bias. The radiographic analysis was conducted systematically while encompassing the evaluation of nine parameters, as described by Dowsey et al. [11]. First, global severity of OA of the tibiofemoral joint was graded according to K&L on the a.p. view of the knee. Osteophytes, periarticular ossicles, joint space narrowing, sclerosis of the subchondral bone, pseudocystic areas with sclerotic walls, and the altered shape of bone ends were used as radiological features for classification of the OA. According to the classification, osteoarthritis was rated as grade 0 (no OA), grade 1 (doubtful OA), grade 2 (minimal OA), grade 3 (moderate OA), and grade 4 (severe OA) using [17]. Afterwards, four distinct radiographic characteristics were assessed on the anteroposterior (a.p.) view of the knee following the guidelines provided by the OARSI atlas. These features included the narrowing of the medial and lateral joint spaces, graded on a scale from 0 to 3, as well as the presence of medial and lateral osteophytes, also graded on a scale from 0 to 3 [18]. Next, subchondral bone loss of the medial and lateral compartment was graded according to the modified Ahlbäck approach classifying attrition on a scale of 0–3 (0 = no attrition, 1 = attrition of doubtful significance [<5 mm], 2 = definite attrition of a moderate degree [5–10 mm], 3 = severe attrition [>10 mm]) [19]. Furthermore, the degree of joint space narrowing in the patellofemoral joint was assessed on the skyline view radiograph of the patella using the grading scale of 0 to 3 specified in the OARSI atlas. Finally, the mechanical axis of the leg was determined using the hip–knee–ankle angle (HKA) on the long leg a.p. view. Therefore, the angle between a line connecting the center of the femoral head with the center of the knee defined as the midpoint of the tibial spines, halfway between the femoral intercondylar notch and the line connecting the center of the knee to the center of the ankle, was measured [11]. Finally, all results were unblinded and the inter-/intra-class correlation coefficients were calculated. Divergent measurements for categorical data were identified between reviewer one and two, and a consensus was reached to determine the final measurement. These agreed-upon radiographic measurements were subsequently utilized for further data analysis.

### 2.4. Statistical Analysis

A sample size calculation was performed for the correlation analysis a priori (Spearman’s rho) assuming a weak, minimum relevant correlation coefficient of 0.25 between OKS and K&L. The power calculation indicated that a minimum of 135 patients would be needed in the study group to provide sufficient power (a = 0.05, b = 0.8) [20]. To minimize selection bias, we incorporated all 138 eligible patients from the year 2019 into the study cohort. The initial question was evaluated by computing Spearman’s rank correlation coefficient for the variables OKS, VAS pain, ROM, and the nine standardized radiographic parameters. The second question was examined through a multiple linear regression analysis to evaluate the correlation among the parameters in a multivariate model, thus aiming to minimize the impact of potential confounding factors. The Oxford Knee Score (OKS) was chosen as the dependent continuous variable, and the analysis was adjusted for BMI, ASA score, pain in the contralateral knee, and the nine radiographic measurement parameters mentioned earlier. To compare continuous variables between groups, the Mann–Whitney U test was employed. Inter-observer reliabilities were computed for all 138 data sets, while intra-observer reliabilities were determined for 14 randomly selected data sets, considering both ordinal and absolute values. This was achieved by calculating the average-measure intra-class correlation coefficients (ICCs) using a two-way random effects model for consistency. To ensure objectivity, repeated measurements for intra-observer reliability were conducted in a blinded manner on two separate occasions (day 1 and day 7) [21,22]. After conducting exploratory data analysis, a Kolmogorov–Smirnov test was employed to evaluate the variables for normal distribution. Among the variables tested, only OKS met the criteria for normal distribution. Consequently, non-parametric tests were utilized. Significance was determined by considering *p*-values below 0.05. Mean values accompanied by standard deviations were used to express continuous variables. Statistical analyses were conducted using SPSS software (version 25.0; IBM Inc., Armonk, New York, NY, USA).

## 3. Results

The inter- and intra-observer intra-class correlation coefficients (ICCs) for radiographic measurements were rated as good to excellent (0.6–1.0). Regarding our first inquiry of the study, both the Oxford Knee Score (OKS) and visual analog scale (VAS) pain score exhibited a weak correlation with the K&L grade, suggesting that patients with more severe radiographic osteoarthritis experienced poorer OKS outcomes and increased pain levels (r = −0.289, *p* = 0.001; r = 0.258, *p* = 0.002).

The absolute value of the passive ROM of the knee is weakly and inversely correlated with the severity of joint space narrowing (r = −0.331, *p* < 0.001) and osteophyte size (r = −0.337, *p* < 0.001) in the lateral tibiofemoral compartment, corresponding to an extension and maximum passive flexion deficit (r = 0.314, *p* < 0.001; r = −0.290, *p* = 0.001, respectively) (r = −0.290, *p* = 0.001; r = −0.303, *p* < 0.001, respectively) (Supplementary Materials).

In relation to our second initial question, analysis through multiple linear regression revealed that two factors, the severity of radiographic tibiofemoral OA based on the K&L scale (r = −4.528, *p* = 0.021) and the radiographic narrowing of the patellofemoral joint space (r = −2.211, *p* = 0.038), were identified as independent variables associated with a poorer OKS (Table 2).

**Figure 2:**
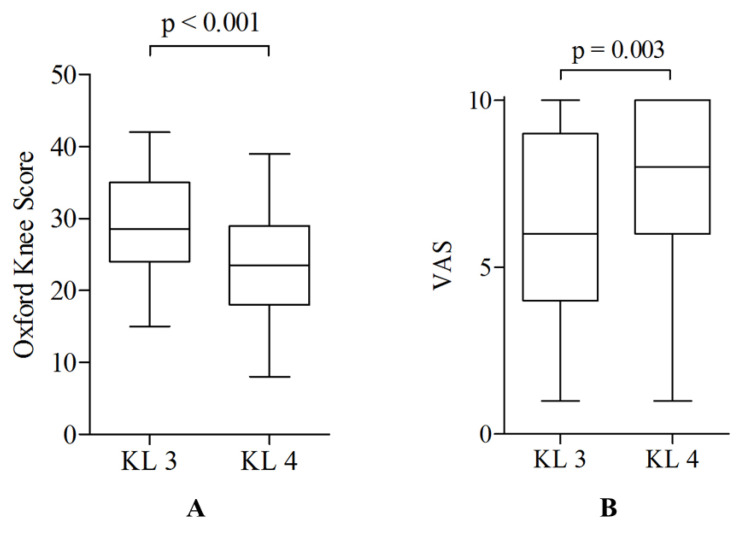
Box plots comparing the (**A**) Oxford Knee Score and (**B**) the VAS pain score between patients with K&L grade 3 and 4 knee osteoarthritis. The boxes show the inter-quartile range (IQR), with the median represented by the band. The whiskers extend to the lowest and highest data points within 1.5 IQR of the lower and upper quartiles following the Tukey boxplot method. The level of significance was set at *p* < 0.05.

Patients with K&L grade 4 osteoarthritis of the knee reported clinically meaningful worse results for the OKS (∆OKS 5.5 points) and higher pain scores (∆VAS 1.7 points) (Figure 2) compared to patients with K&L grade 3, with each difference being only slightly above the threshold for the minimum clinically important difference (MCID).

**Figure 3:**
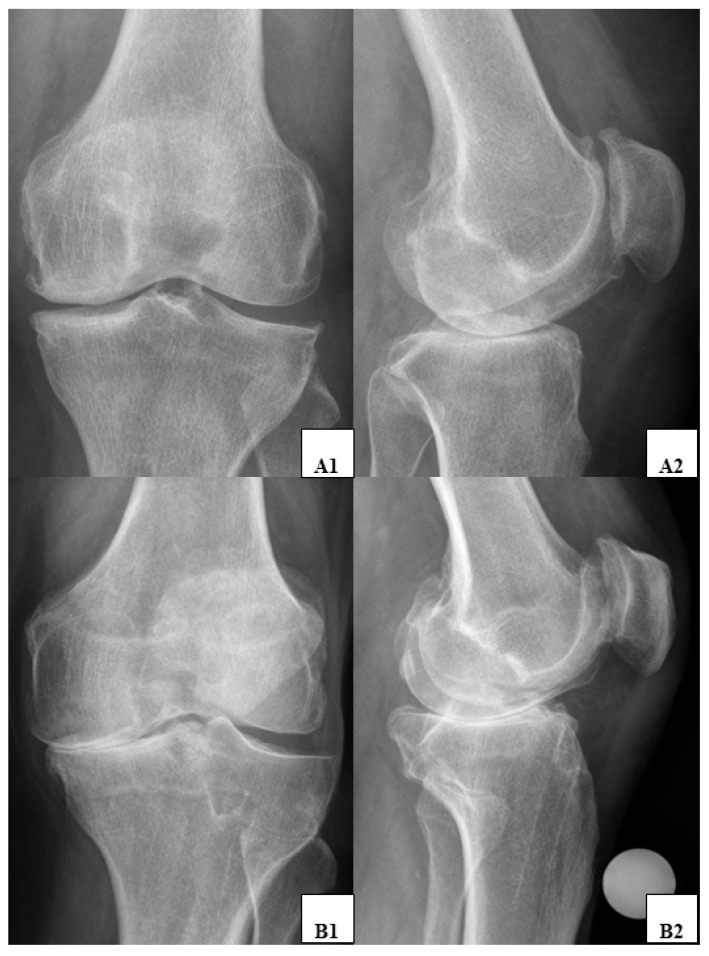
Exemplary a.p. and lateral knee radiographs from two different patients are shown. Patient A (**A1**,**A2**) exhibited poor clinical function (OKS = 15, ROM 0–10–95°) despite only moderate OA according to radiographic criteria (K&L grade 3). Patient B (**B1**,**B2**), on the other hand, showed good clinical function (OKS = 36, ROM 0–0–130°) despite severe OA according to radiographic criteria (K&L grade 4).

The passive ROM did not reveal any significant clinical difference between patients classified as K&L grade 3 or 4 (Table 3). Figure 3 presents two radiographic examples of two patients, with patient A showing poor clinical function despite less severe radiographic findings and patient B showing good clinical function despite severe radiographic osteoarthritis.

## 4. Discussion

The main findings and answers to our study questions can be summarized as follows: Radiographic severity of osteoarthritis (OA) in the tibiofemoral and patellofemoral joints was found to be independently associated with a worse Oxford Knee Score (OKS). However, the correlations between subjective clinical outcome measures (OKS, VAS pain score), objectively measured range of motion (ROM), and radiographic OA severity were weak and had limited clinical significance. This paper focuses on investigating the associations between subjective (pain and function measured with the OKS) and objective clinical findings (range of motion) and radiographic degenerative structural changes in patients with advanced knee osteoarthritis, an area that remains incompletely understood. Therefore, it is important to interpret the present results in the context of the limited and sometimes contradictory existing literature.

Odding et al. showed significant associations for locomotor disability and pain, comparable to the associations found between locomotor disability and severe radiographic osteoarthritis (K&L ≥ 3) with pain [23]. Consequently, they proposed that joint pain has a greater impact on locomotor disability than radiographic osteoarthritis alone. Duncan et al. reported associations between individual WOMAC items for pain and disability pertaining to weight-bearing mobility and radiographic osteoarthritis according to K&L, which compare well to our findings of weak but significant correlations for worse PROMs (pain and OKS) with higher K&L grades [10]. However, both of these later studies did not include knee ROM as an objective clinical outcome parameter, patellofemoral joint radiographs, or detailed radiographic measurements according to the Ahlbäck criteria. In contrast, different studies found no associations between disability and severe radiographic knee osteoarthritis when controlling for other variables such as age, sex, and BMI [11,24]. We seized the suggestion by Duncan et al., “to consider the severity and compartmental involvement of radiographic osteoarthritis and its relationship with symptoms” [10]. Considering this, the OKS showed a correlation in our study group with overall radiographic osteoarthritis, as measured by K&L and patellofemoral joint space narrowing. However, it did not correlate with scores for isolated medial or lateral compartment joint space narrowing. Previous studies have reported discrepancies between the severity of global radiographic OA and functional impairment in patients with knee OA [11,25]. In contrast, several reported a consistent association between the severity of pain, stiffness, physical function, and the presence of radiographic osteoarthritis [8,10,23,26,27,28]. Additionally, Szebenyi et al. reported a weak association between function and combined tibiofemoral and patellofemoral joint space narrowing [29]. Our study confirms the results of the previous two studies. Patients with K&L grade 4 OA had notably higher levels of pain compared to those with grade 3 OA (VAS 6.1 vs. 7.8) and also had significantly poorer OKS scores (23.7 vs. 29.2), both slightly surpassing the MCID threshold (VAS: 1.4 points; OKS: 5 points [30,31]). However, we have to acknowledge that several patients with K&L grade 4 knee OA may have had no pain at all [12].

In terms of the relationship between osteophytes and pain, three studies found high sensitivity but low specificity (82.5% and 23.3%, respectively) [31,32,33]. Previous research has shown a correlation between osteophyte size, knee pain, and clinical function [11,34,35,36]. Our study partially supports these findings, as we observed a correlation between the maximum flexion deficit, lateral compartment joint space narrowing, and osteophyte size. However, it remains unclear whether lateral osteophytes are the cause of pain and functional impairment or simply an indicator of advanced osteoarthritis [11].

### 4.1. Limitations of the Study

The current study has limitations. The use of a single center for the study design may introduce potential selection bias limiting the generalizability of the results to the broader population of patients with advanced knee osteoarthritis. To minimize this effect, we identified a consecutive cohort of patients with advanced primary osteoarthritis of the knee who visited our outpatient clinic over a one-year period. However, a significant number of patients had to be excluded due to incomplete radiographs, mainly involving the patellofemoral joint.

Furthermore, co-treatment bias cannot be excluded due to treatment with analgesic drugs or physiotherapy potentially biasing the subjective and objective clinical parameters. However, it is important to note that it is common practice at our arthroplasty center for patients to have undergone at least three months of physiotherapy and to have used non-steroidal anti-inflammatory drugs as pain killers prior to joint replacement.

Lastly, we recognize a potential observer bias during measurements of the objective ROM of the knee with a goniometer. We tried to reduce this effect by assessing ROM in 5-degree increments as the minimal interval.

### 4.2. Strengths of the Study

Despite the limitations mentioned above and the fact that several studies have investigated the association of radiographic knee OA, pain, and clinical function, our study has notable strengths and provides additional knowledge to the reader. Bedson et al. proposed three specific reasons for the discordance between X-rays and symptoms [33], and we addressed these reasons in our study design. Firstly, we used multiple weight-bearing X-ray views of the knee, including the patellofemoral joint, which is not extensively covered in the literature. In our study, we have provided information on radiographs of the patellofemoral joint and long leg axis, which are particularly underrepresented in the existing literature on this topic. Secondly, we differentiated pain and disability using the visual analogue scale (VAS) and the validated OKS as PROMs. This is an important point. Most studies investigated their patients using the WOMAC score, which consists of three subscales (pain, stiffness/ROM, and clinical function) where the information for all three is provided by the patient. In our study, the information on stiffness and ROM was collected by a medical doctor. Thus, we collected more objective data on ROM for further analysis. Thirdly, our study population closely matched a reference population with advanced knee osteoarthritis from the Swedish Knee Arthroplasty Register in terms of gender, patient age, ASA score, and BMI. However, our study did reveal a slight shift towards a higher percentage of ASA III scores and BMI values for obesity class I and II. The pain scores in our study were slightly higher at 7.5 compared to 6.1 in the reference population, but these values are still below the threshold for the MCID. Fourthly, our study had sufficient statistical power, as demonstrated by the sample size calculation, which is rarely reported in studies on this topic. Lastly, all radiographic measurements were independently performed by two reviewers, and any discrepancies were resolved through consensus. With good to excellent inter-rater reliability values, we can confidently assert the high quality of our radiographic measurements.

## 5. Conclusions

In conclusion, numerous studies have explored the correlation between pain, function, and radiographic grade in knee OA. Our study makes distinctive contributions by providing additional insights to the current body of literature. We objectively assessed ROM and conducted a detailed radiographic evaluation that incorporated rarely studied radiographic views and parameters. Moreover, our study cohort was appropriately powered, allowing us to draw the following conclusions: Pain, patient-reported outcomes, and limited range of motion demonstrated a weak correlation with the severity of advanced knee osteoarthritis as seen on radiographs. The decrease in range of motion shows only a weak association with the severity of advanced knee osteoarthritis, which is in contrast to what has been reported for hip osteoarthritis in the literature. This has implications when radiographs alone are used to evaluate the severity of OA in legal cases, particularly in countries like Germany. The radiographic severity of OA does not accurately reflect the level of pain, loss of function, and reduced range of motion. Consequently, it may underestimate or overestimate the compensation that a patient should receive. Therefore, it is necessary to consider more detailed information when discussing such cases. Furthermore, the OKS should not be used as the sole screening tool to determine a patient’s eligibility for total knee arthroplasty before scheduling an appointment at an arthroplasty clinic. Relying solely on the OKS may lead to the under- or overidentification of patients who may potentially benefit from knee arthroplasty. It is important to note that patients with better preoperative function are less likely to experience significant functional improvement after knee arthroplasty [37]. Therefore, our study highlights the importance of considering patients’ self-reported outcomes when advising individuals with advanced knee osteoarthritis, who may be potential candidates for knee arthroplasty and not only radiographic imaging.

## Figures and Tables

**Table 1:** Patient demographics and clinical parameters. R = right; L = left; *n* = number of items; SD = standard deviation.

Variable	
Side (R:L) (*n*)	78:60
Gender (F:M) (*n*)	80:58
Age (years) (mean ± SD)	65.2 ± 10.2
Body mass index (kg/m^2^) at surgery (mean ± SD)	31.0 ± 5.8
Normal (*n*)	27
Overweight (*n*)	36
Obese Class I (*n*)	47
Obese Class II (*n*)	16
Obese Class III (*n*)	12
ASA score (I/II/III/IV) (*n*)	14/73/51/0
VAS knee pain (0–10) (mean ± SD)	7.4 ± 2.3
VAS contralateral knee pain (0–10) (mean ± SD)	3.4 ± 2.8
Passive knee extension deficit (degree) (mean ± SD)	2.7 ± 4.4
Maximum passive flexion of the knee (degree) (mean ± SD)	117.2 ± 15.0

**Table 2:** Multiple linear regression analysis to assess the impact on Oxford Knee Score (r^2^ = 0.214). The level of significance was set at *p* < 0.05 and, if achieved, is written in bold and marked with an asterisk (*).

Model	Unstandardized β Coefficients	Standard Error	Standardized β Coefficients	*p*-Value
(constant)	52.394	6.138	-	-
BMI	−0.160	0.111	−0.127	0.153
ASA Score	−1.406	1.001	−0.122	0.163
Pain (VAS) contralateral knee	−0.340	0.226	26	0.135
K&L tibiofemoral	−4.528	1.939	−0.257	0.021 *
Medial joint space narrowing	−0.759	1.708	−0.082	0.657
Lateral joint space narrowing	−1.108	0.831	−0.170	0.185
Medial osteophyte	0.723	1.007	0.073	0.474
Lateral osteophyte	1.059	1.104	0.120	0.340
Medial attrition score	0.061	1.218	0.007	0.960
Lateral attrition score	2.597	1.928	0.196	0.180
Patellofemoral joint space narrowing	−2.211	1.052	−0.177	0.038 *
Long leg axis	0.154	0.163	0.186	0.347

**Table 3:** Comparison of VAS pain, ROM, and OKS in individuals with K&L grade 3 and 4 osteoarthritis of the knee. The level of significance was set at *p* < 0.05 and, if achieved, is written in bold and marked with an asterisk (*).

Variable	K&L Grade 3 (*n* = 30)	K&L Grade 4 (*n* = 108)	*p*-Value
VAS pain (mean ± SD and range)	6.1 ± 2.8 (1–10)	7.8 ± 2.1 (1–10)	0.003 *
Knee extension deficit in degree (mean ± SD)	1 ± 3 (0–10)	3 ± 5 (0–20)	0.048 *
Maximum knee flexion in degree (mean ± SD)	122 ± 14 (90–140)	116 ± 15 (85–140)	0.052
Oxford Knee Score (mean ± SD)	29.2 ± 6.9 (15–42)	23.7 ± 6.9 (8–39)	<0.001 *

## Data Availability

The data presented in this study are available on request from the corresponding author. The data are not publicly available due to patients’ data protection rights.

## Supplementary Materials

The following supporting information can be downloaded at: https://www.mdpi.com/article/10.3390/jcm12165420/s1. Table S1: Spearman-Rho correlation coefficients between clinical and radiographic outcome measurements. Level of significance was set at *p* < 0.05 and, if achieved, is written in bold letters, and marked with an asterisk (*). In case of *p* < 0.001 correlation is assumed to be highly significant and is marked with two asterisks (**).
