# Supplementary material for: What Is the Correlation between Clinical and Radiographic Findings in Patients with Advanced Osteoarthritis of the Knee?
Source: J Clin Med. 2023 Aug 21;12(16):5420. doi: 10.3390/jcm12165420 (PMC10455573; doi:10.3390/jcm12165420)
Supplement: Supplementary file 1 [file jcm-12-05420-s001.zip › jcm-2491452-supplementary.pdf]

Supplementary Material.

Table S1. Spearman-Rho correlation coefficients between clinical and radiographic outcome measurements. Level of significance was set at $p < 0.05$ and, if achieved, is written in bold letters, and marked with an asterisk (*). In case of $p < 0.001$ correlation is assumed to be highly significant and is marked with two asterisks (**).

**Variable**	Pain (VAS)	OKS	Knee extension deficit in °	Maximum flexion of the knee in °	ROM absolute value in °	BMI	K&L tibiofemoral	Medial joint space narrowing	Lateral joint space narrowing	Medial osteophyte	Lateral osteophyte	Medial attrition	Lateral attrition	Patellofemoral joint space narrowing	Long leg axis in °
Pain (VAS)	1	-	-	-	-	-	-	-	-	-	-	-	-	-	-
Oxford Knee Score	**-0.648****	1	-	-	-	-	-	-	-	-	-	-	-	-	-
Knee extension deficit in °	**0.209***	**-0.187***	1	-	-	-	-	-	-	-	-	-	-	-	-
Max. flexion of the knee in °	**-0.307****	**0.229****	**-0.352****	1	-	-	-	-	-	-	-	-	-	-	-
ROM absolute value in °	**-0.324****	**0.248****	**-0.529****	**0.977****	1	-	-	-	-	-	-	-	-	-	-
BMI	0.122	**-0.187***	0.085	**-0.285****	**-0.276****	1	-	-	-	-	-	-	-	-	-
K&L tibiofemoral	**0.258****	**-0.289****	0.169*	-0.167	**-0.184***	0.135	1	-	-	-	-	-	-	-	-
Medial joint space narrowing	0.142	**-0.207***	0.068	-0.038	-0.059	**0.191***	**0.526****	1	-	-	-	-	-	-	-
Lateral joint space narrowing	0.016	-0.030	**0.314****	**-0.290****	**-0.331****	-0.007	-0.031	**-0.444****	1	-	-	-	-	-	-
Medial osteophyte	0.107	-0.075	**0.211***	-0.144	**-0.180***	**0.287****	0.117	**0.368****	0.105	1	-	-	-	-	-
Lateral osteophyte	0.013	-0.016	**0.290****	**-0.303****	**-0.337****	**0.181***	0.029	**-0.311****	**0.717****	**0.228****	1	-	-	-	-
Medial attrition	0.089	-0.043	0.073	-0.044	-0.052	**0.172***	**0.217***	**0.684****	**-0.287****	**0.444****	-0.124	1	-	-	-
Lateral attrition	-0.086	0.071	0.107	-0.147	-0.144	-0.097	0.033	**-0.633****	**0.573****	**-0.177***	**0.439****	**-0.515****	1	-	-
Patellofemoral joint space narrowing	0.148	**-0.251****	0.136	-0.136	-0.163	0.095	0.112	0.089	0.151	0.150	**0.168***	0.036	0.070	1	-
Long leg axis in °	-0.010	0.010	-0.038	0.076	0.075	0.134	**0.186***	**0.709****	**-0.424****	**0.350****	**-0.264****	**0.791****	**-0.663****	-0.119	1
